# Supplementary material for: Anisotropic Thermally Conductive Perfluoroalkoxy Composite with Low Dielectric Constant Fabricated by Aligning Boron Nitride Nanosheets via Hot Pressing
Source: Polymers (Basel). 2019 Oct 10;11(10):1638. doi: 10.3390/polym11101638 (PMC6835865; doi:10.3390/polym11101638)
Supplement: Supplementary file 1 [file polymers-11-01638-s001.docx]

Anisotropic Thermally Conductive Perfluoroalkoxy Composite with Low Dielectric Constant Fabricated by Aligning Boron Nitride Nanosheets via Hot Pressing

Xinru Zhang ^1,2,†^, Xinzhi Cai ^1,†^, Xiaoyu Xie ^1^, Changyu Pu ^1^, Xuanzuo Dong ^1^, Zeyi Jiang ^1,3,^*,
Ting Gao ^1^, Yujie Ren ^4^, Jian Hu ^5^ and Xinxin Zhang ^1,3^

^1^ School of Energy and Environmental Engineering, University of Science and Technology, Beijing 100083, China; xinruzhang@ustb.edu.cn (X.Z.); b20170070@xs.ustb.edu.cn (X.C.); xiexiaoyuustb@163.com (X.X.); nmcfpcy@163.com (C.P.); 15901217705@163.com (X.D.); gn740924@me.ustb.edu.cn (T.G.); xxzhang@ustb.edu.cn (X.Z.)

^2^ Beijing Engineering Research Center of Energy Saving and Environmental Protection, University of Science and Technology, Beijing 100083, China

^3^ Beijing Key Laboratory for Energy Saving and Emission Reduction of Metallurgical Industry, University of Science and Technology, Beijing 100083, China

^4^ China Energy Conservation and Environmental Protection Group, Beijing 100082, China; renyujie@cecep.cn

^5^ China Energy Conservation and Environmental Protection Group, National Machinery United Electric Power (Ningxia) Co., Ltd., Yinchuan 750011, China; huazhonghujian@163.com

***** Correspondence: zyjiang@ustb.edu.cn; Tel.: +86-10-62334971

† These authors contributed equally to this work.

**S1. Characterization of BN bulk material**


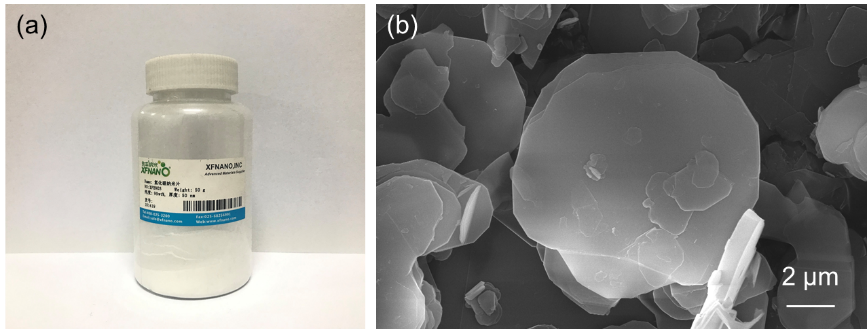


**Figure S1.** (**a**) The used BN bulk material. (**b**) SEM of BN.

**S2. Exfoliating BN in a series of ethanol–water mixtures**

On the basis of surface thermodynamics, the solvents property would significantly affect the exfoliation of nanosheets [1-3].Therefore, before experiment, we studied exfoliation and dispersion of BN in a series of ethanol–water mixtures. As shown in Figure S2, the concentrations of BNNs, which were evaluated by optical density, depended strongly on the volume fraction of ethanol, and the highest concentration of BNNs was obtained in 65 vol % ethanol–water mixture. Accordingly, in this study, the BN bulk material was exfoliated in 65 vol % ethanol–water mixture.


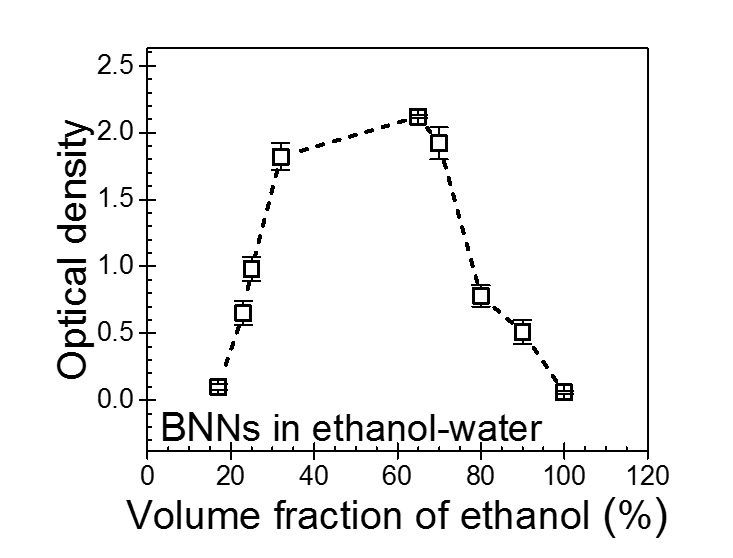


**Figure S2.** Exfoliating BN in a series of ethanol–water mixtures.

**S3. Frequency versus *θ***


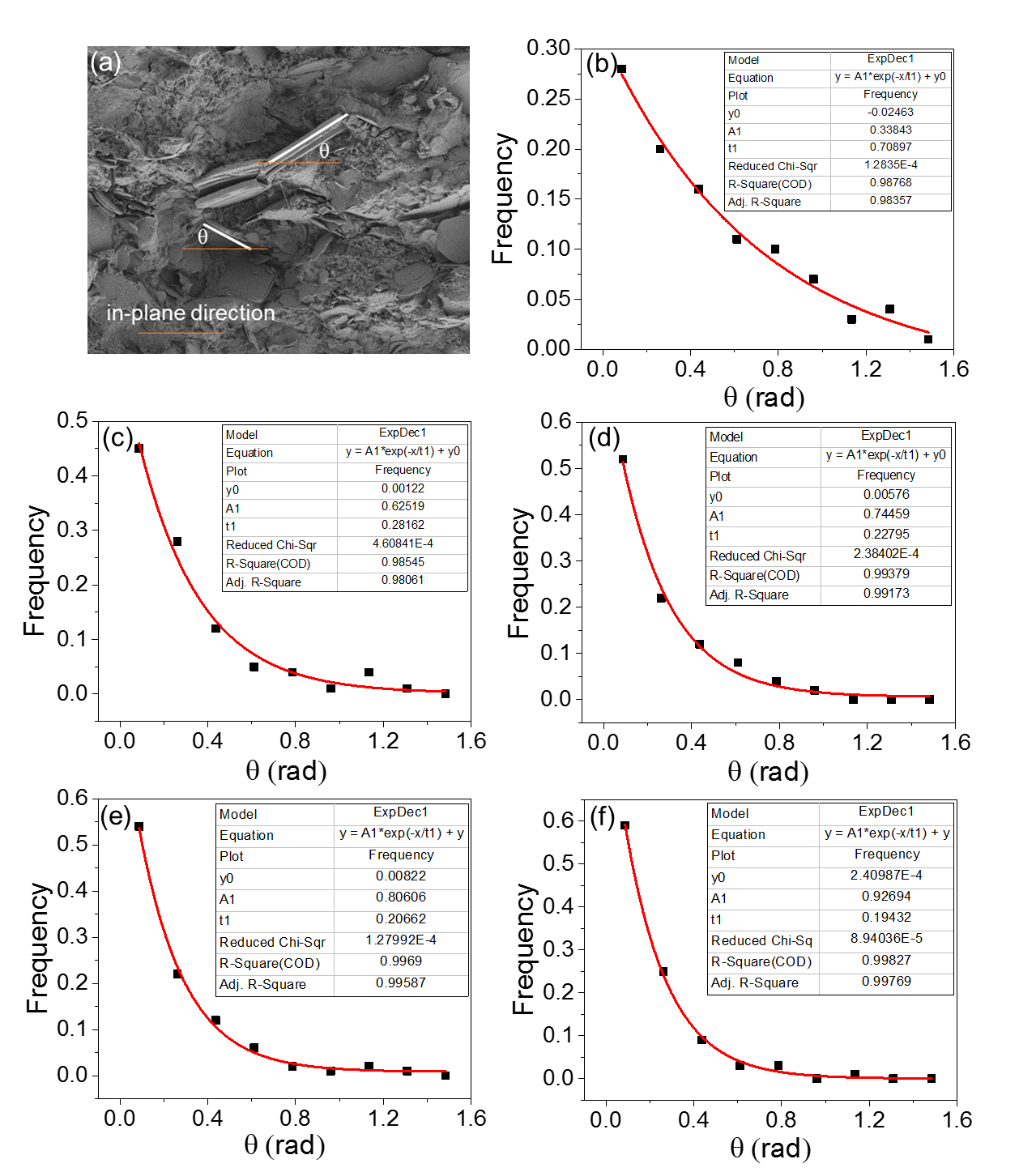


**Figure S3.** (a) Illustration of θ. (b–f) Frequency versus *θ* from the SEM images of PFA composites containing 10, 15, 20, 25 and 30 wt% BNNs.

**Table S1.** The parameters used in the EMT model.

| Parameters | BNNs | PFA |
| --- | --- | --- |
| Size (nm) | 2000 |  |
| Thickness (nm) | 5.6 |  |
| In-plane thermal conductivity (W m^-1^·K^-1^) | 390 [4] | 0.26 |
| Through-plane thermal conductivity (W m^-1^·K^-1^) | 2 [4] | 0.26 |
| Density (kg/m^3^) | 1.96 | 2.23 |

**Table S2.** The heat resistance index (THRI) for pure PFA and the BNNs–PFA composites.

| Samples | Weight loss temperature (℃) | | The heat resistance index（THRI）  (℃) |
| --- | --- | --- | --- |
|  | T_5_ | T_30_ |  |
| Pure PFA | 540.9 | 575.6 | 275.2 |
| 1% BNNs | 524.6 | 562.5 | 268.2 |
| 5% GNs | 535.7 | 563.6 | 270.7 |
| 10% GNs | 527.4 | 563.4 | 269.0 |
| 15% GNs | 530.9 | 567.3 | 270.8 |
| 20% GNs | 539.3 | 568.0 | 272.7 |
| 25% GNs | 531.7 | 566.1 | 270.6 |
| 30% GNs | 529.2 | 569.9 | 271.3 |

Note: T_5_ and T_30_ are the temperature at 5% and 30% weight loss, respectively.

**References**

1. Cunningham, G.; Lotya, M.; Cucinotta, C.S.; Sanvito, S.; Bergin, S.D.; Menzel, R.; Shaffer, M.S.P.; Coleman, J.N. Solvent Exfoliation of Transition Metal Dichalcogenides: Dispersibility of Exfoliated Nanosheets Varies Only Weakly between Compounds. ACS nano 2012, 6, 3468-3480.

2. Chhowalla, M.; Shin, H.S.; Eda, G.; Li, L.J.; Loh, K.P.; Zhang, H. The chemistry of two-dimensional layered transition metal dichalcogenide nanosheets. Nature chemistry 2013, 5, 263-275.

3. Hernandez, Y.; Nicolosi, V.; Lotya, M.; Blighe, F.M.; Sun, Z.; De, S.; Mcgovern, I.T.; Holland, B.; Byrne, M.; Gun'Ko, Y.K. High-yield production of graphene by liquid-phase exfoliation of graphite. Nature nanotechnology 2008, 3, 563-568.

4. I. Jo, M.T. Pettes, J. Kim, K. Watanabe, T. Taniguchi, Z. Yao, L. Shi, Thermal conductivity and phonon transport in suspended few-layer hexagonal boron nitride, Nano letters 13(2) (2013) 550-4.
